# Quality by design approach for development and characterization of gabapentin-loaded solid lipid nanoparticles for intranasal delivery: *In vitro, ex vivo*, and histopathological evaluation

**DOI:** 10.22038/IJBMS.2024.76281.16511

**Published:** 2024

**Authors:** Mahmut Ozan Toksoy, Fırat Aşır, Mert Can Güzel

**Affiliations:** 1 Department of Pharmaceutical Technology, Dicle University, Diyarbakır, Turkey; 2 Department of Histology and Embryology, Dicle University, Diyarbakır, Turkey; 3 Department of Pharmaceutical Technology, Ege University, İzmir, Turkey

**Keywords:** Box-Behnken design, Gabapentin, Histopathology, Nasal delivery, Permeation, Release kinetics, Solid lipid nanoparticle

## Abstract

**Objective(s):**

”Quality by Design” (QbD) is a novel approach to product development that involves understanding the product and process, as well as the relationship between critical quality attributes (CQA) and critical process parameters (CPP). This study aimed to optimize the gabapentin-loaded solid lipid nanoparticle formulation (GP-SLN) using a QbD approach and evaluate in vitro and ex vivo performance.

**Materials and Methods:**

The GP-SLN formulation was created using the microemulsion method by combining Gelucire 48/16, Tween 80, and Plurol Oleique CC 497. The Box-Behnken experimental design was adopted to investigate the effects of independent factors on dependent factors. The GP-SLN formulation was assessed based on particle size and distribution, zeta potential, morphology, entrapment efficiency, release kinetics, permeation parameters, stability, and nasal toxicity.

**Results:**

The nanoparticles had a cubical shape with a particle size of 185.3±45.6 nm, a zeta potential of -24±3.53 mV, and an entrapment efficiency of 82.57±4.02%. The particle size and zeta potential of the GP-SLNs remained consistent for 3 months and followed Weibull kinetics with a significantly higher *ex vivo* permeability (1.7 fold) than a gabapentin solution (GP-SOL). Histopathology studies showed that intranasal administration of the GP-SLN formulation had no harmful effects.

**Conclusion:**

The current study reports the successful development of a GP-SLN formulation using QbD. A sustained release of GP was achieved and its nasal permeability was increased. Solid lipid nanoparticles with optimum particle size and high entrapment efficiency may offer a promising approach for the intranasal delivery of drugs.

## Introduction

Epilepsy is a neurological disorder characterized by sudden and repetitive electrical discharges such as seizures, convulsions, and involuntary movements. It is estimated that more than 50 million people worldwide suffer from epilepsy (1). Unfortunately, epilepsy patients often face discrimination, misunderstanding, and social stigma. In addition, they experience stress in their daily lives due to seizures (2-5). 

Gabapentin (GP), an analog of gamma-aminobutyric acid (GABA), is used in the treatment of epilepsy. GP contains carboxylic acid and primary amine, with pKa values of 3.68 and 10.7, respectively. However, GP is classified as a BCS Class III drug, and absorption problems are often observed. Although the oral bioavailability of a 300 mg dose of GP is 60%, bioavailability gradually decreases as the dose increases (6, 7).

Administering drugs via different methods significantly affects bioavailability, side effects, and targeted delivery. Invasive methods are considered to be more effective because of their rapid response and site-specific delivery, but they require skill and come with safety concerns. On the other hand, it may be necessary to administer drugs orally at high doses to maintain sufficient concentration due to first-pass metabolism (8). Intranasal delivery is another non-invasive method that is convenient and safe as patients can easily self-administer the drugs, as such intranasal delivery is a practical approach to drug administration (9).

The nasal cavity contains a rich network of vascularity that helps drugs avoid first-pass metabolism. This allows for rapid and more efficient drug absorption. Additionally, delivering drugs nasally can be an effective way to target specific sites in the body (10) and there have been many studies that indicate intranasal drug delivery can also be a useful and effective method to target drugs to the brain. Recent research has shown that nano-drug delivery systems are more effective in transporting drugs from the nose to the brain compared to traditional oral drug solutions (11, 12). In addition, some studies suggest that intranasal drug delivery can result in higher concentrations of drugs in the brain than intravenous drug delivery (13, 14).

Using lipid-based drug delivery systems can be an effective way to enhance the absorption of drugs through the nasal cavity (15, 16). Solid lipid nanoparticles (SLNs) are nano-sized (50–1000 nm), spherical particles with a solid core matrix made up of lipids that are biodegradable and biocompatible and remain solid at room temperature. Hydrophilic or lipophilic drugs can be dissolved or dispersed in the solid lipid core matrix. Due to their unique properties, such as their large surface area, and high drug loading capacity, SLNs are an ideal carrier for improving the therapeutic effects of drugs (17).

The Food and Drug Administration (FDA) has adopted Quality by Design (QbD), which is a new approach to product development. This strategy involves understanding the product and process, as well as the relationship between the critical quality attributes (CQA) and critical process parameters (CPP), to establish a controlled design space. By doing so, a high-quality product can be produced that would reduce the total amount of waste (time, money, and material) generated during the production process (18).

To ensure a high-quality product, QbD assesses both the product and the process. According to the International Conference on Harmonization (ICH Q8), QbD involves every step of pharmaceutical process development, resulting in a comprehensive understanding of both the product and the process (19). Researchers have developed various statistical designs, such as the Box-Behnken design (BBD), Taguchi, D-Optimal, Placket Burman, and 2-level factorial, using specialized design tools. BBD is a very popular choice for researchers in the development of new formulations, as it requires less time and suggests fewer iterations. Additionally, BBD helps to identify the best formulation to achieve the intended goal by highlighting the impact of independent factors and variables on dependent factors (20).

A study was conducted to investigate the effectiveness of gabapentin loaded into solid lipid nanoparticles (21) in which different formulations were developed and characterized. However, traditional experimental designs have several drawbacks, as changing one factor at a time while keeping others constant results in a large number of experiments, which takes a lot of time and money. Moreover, it is not possible to examine the interactions of factors with each other using this approach. Furthermore, the study did not examine the effects of independent variables on the formulation. Experimental designs, however, provide more accurate results with fewer experiments than the traditional approach when developing formulations (22).

In our research, we employed Gelucire 48/16 as a solid lipid due to its unique properties. Unlike other derivatives, Gelucire 48/16 is made up of polyethylene glycol-32 (PEG-32) stearate and has a high hydrophilic-lipophilic balance (HLB) value that may enhance the solubility of hydrophilic drugs in the carrier system, leading to improved miscibility and dispersibility (23, 24). Tween 80 is a non-ionic surfactant that also has a high HLB value and is commonly used as a surfactant in lipid nanocarrier systems (25, 26). However, the non-ionic properties of Tweens may result in inadequate repulsive forces between particles. Therefore, it should be used together with co-surfactants to stabilize the interfaces of lipid particles. Plurol Oleique CC 497 is one of the most commonly used cosurfactants (27, 28).

This study aimed to create and optimize a formulation of gabapentin-loaded solid lipid nanoparticles (GP-SLN) for nasal administration. The effects of various independent variables on the formulation were examined using BBD. The characteristics of the GP-SLN formulation, particle size, zeta potential, polydispersity index, entrapment efficiency, and stability were investigated. The GP-SLN formulation and a standard gabapentin solution (GP-SOL) were comprehensively evaluated to determine the release kinetics, permeability, and flux of gabapentin. Additionally, histopathological studies were conducted on sheep nasal mucosa.

## Materials and Methods

***Materials***

Gabapentin was obtained from TCI Chemicals (Japan), Tween 80 was obtained from Merck (Germany), Gelucire 48/16, and Labrasol and Plurol Oleique CC 497 were kindly gifted by Gatte-Fosse (France). Methanol and acetonitrile were purchased from Merck (Germany). Dialysis membrane molecular weight cut-off (mwco: 12000) was obtained from Merck (Germany). All other chemicals used were analytical grade.

***Methods***

***Determination of the gabapentin***

Gabapentin was analyzed using a high-performance liquid chromatography system (HPLC, Shimadzu DGU-20A5R+LC 20 AT+SIL 20 AHT+CTO 10 ASVP+SPD M20A, Japan) using a reverse phase C18 column (5 µm, 250 mm×4.6 mm). The mixture of 0.01 M potassium dihydrogen phosphate buffer and acetonitrile (90:10, v/v) was used as the mobile phase (29, 30). The flow rate was 1 ml/min and the injection volume was 20 µl. The study was carried out at a wavelength of 210 nm. Standard solutions in the simulated nasal fluid (SNF, pH 6.4) were prepared in concentrations of 5-80 µg/ml (31, 32). The study was carried out at room temperature (25 ^°^C).

***Preparation of the gabapentin-loaded solid lipid nanoparticles (GP-SLN)***

The GP-SLN formulation was created using a modified microemulsion method (33), which can be described briefly as follows: Gelucire 48/16 was heated just above its melting point (60 ^°^C). Separately, Plurol Oleique CC 497 and Gabapentin (50 mg) were added to this molten phase to form the oil phase. Tween 80 was added to distilled water and heated to the same temperature as the oil phase in a separate container to form the water phase. Once both phases were at the same temperature (60 ^°^C), the oil phase was combined with the water phase to create a microemulsion. This microemulsion was then poured into distilled water at 4 ^°^C at a 1:50 ratio. The mixture was stirred for 45 min at 900 rpm to successfully produce the GP-SLNs.

***Box-Behnken experimental design***

The Box-Behnken design was employed to evaluate the impact of the ingredients on the formulation. Independent (oil, surfactant, and co-surfactant amounts) and dependent (particle size, polydispersity index, and zeta potential) factors were examined using the Design-Expert 12.0.3 software package (Stat-Ease Inc, Minneapolis, MN, USA). In the BBD model, the design specifies the lowest and highest ratios of substances for 17 formulations prepared in mixed order. Five of these formulations are midpoints to determine experimental error and precision.

The percentage of the independent variables is shown in Table 1. The ratios of Gelucire 48/16 (X_1_, 10-20%), Tween 80 (X_2_, 12-20%), and Plurol Oleique CC 497 (X_3_, 3-7%) were determined based on Gasco’s patent (34). Dependent variables were determined to be at minimal values for particle size (Y_1_), polydispersity index (Y_2_), and maximum value for zeta potential (Y_3_). Factor levels were coded as -1 (low), 0 (medium), and +1 (high) respectively.

The results were suitable for the quadratic model, as expressed by Equation 1.

$Y=\beta_{0}+\beta_{1}X_{1}+\beta_{2}X_{2}+\beta_{3}X_{3}+\beta_{4}{X_{1}}^{2}+\beta_{5}X_{1}X_{2}+\beta_{6}X_{1}X_{3}+\beta_{7}{X_{2}}^{2}+\beta_{8}X_{2}X_{3}+\beta_{9}{X_{3}}^{2}$

Equation 1

According to this, Y was the response, X_1_-X_3_ were independent variables, β_0_ was the intercept, and β_1_-β_9_ gave the regression coefficients. The design was validated by analysis of variance (ANOVA).

***Particle size, polydispersity index, and zeta potential***

Particle size (ps), polydispersity index (PDI), and zeta potential (zp) measurements were performed using a Malvern Zeta Sizer (Malvern Nano ZS, UK). Before the evaluation, the samples were diluted with deionized water in a 1:10 ratio.

***Morphological studies***

The morphological characteristics of the fabricated GP-SLN formulation were examined using a scanning electron microscope (SEM, Dicle University DUBTAM SEM Laboratory). The imaging was performed at 10 kV under low vacuum conditions and with a magnification of 40000-100000. Before the study, the samples were diluted with a 1:1000 ratio in deionized water, dropped onto the grid, and left to dry for 24 hr at room temperature (25 ^°^C).

***Solid state characterization studies***

The thermal properties of the gabapentin, Gelucire 48/16, and a physical mixture of the gabapentin and Gelucire 48/16 were analyzed using a differential scanning calorimeter (DSC-60, Shimadzu, Japan). The samples were compressed and sealed in aluminum pans before being subjected to thermal analysis. The samples were heated from 20 to 250 ^°^C at a rate of 10 ^°^C/min to determine their thermal behavior (35).

Agilent Cary 630 (Agilent, USA) was used to scan gabapentin, Gelucire 48/16, and a combination of the two for Fourier transform infrared spectroscopy (FTIR) analysis in attenuated total reflectance mode. The samples were placed in voids in modest amounts and scanned at a resolution power of 4 cm^-1^ in the infrared range between 400 and 4000 cm^-1^.

***Entrapment efficiency***

The GP-SLNs were centrifuged at 0 ^°^C at 16000 rpm for 30 min. The supernatant was taken and filtered through a 0.22 µm PVDF syringe filter. The amount of free gabapentin was analyzed by HPLC as mentioned above (n=3, mean±SD). The entrapment efficiency (EE%) was calculated using Equation 2.

$\mathrm{EE\%}=G_{0}-G_{1}/G_{0}\times 100$

Equation 2

G_0_ is the total amount of gabapentin in the formulation, and G_1_ is the amount of free gabapentin.

***In vitro release studies***

In this study, a dialysis bag (mwco: 12000) was cut to fit Franz diffusion cells and was kept in SNF before use. Donor and receptor chambers were then filled with SNF. The gabapentin solution (GP-SOL) and the GP-SLN formulation (each containing 50 mg of gabapentin) were loaded into the donor cell. Studies were carried out at 37 ^°^C with a continuous stir at 50 rpm. Samples (1 ml) were collected at specified time points (0, 0.25, 0.5, 0.75, 1, 2, 3, 4, 6, and 8 hr) and an equal volume of fresh SNF was added to maintain sink condition (n=3, mean±SD).

***Release kinetics study***

*In vitro* release profiles of GP-SOL and the GP-SLN formulation were assessed with the DDSolver software. Several mathematical models (Zero-order, First-order, Higuchi, Korsmeyer-Peppas, Peppas-Sahlin, Baker-Lonsdale, Hopfenberg, and Weibull) were applied to evaluate the GP release kinetics from the SLNs. Based on the *in vitro* release data, the DDSolver program was used to determine the Akaike information criterion (AIC), coefficient of determination (r^2^), adjusted coefficient of determination (r^2^ adjusted), and the model selection criterion (MSC) for the selection of the “best fit” models (36).

***Ex vivo permeation studies***

Sheep nasal mucosa, obtained from a slaughterhouse, was cleaned and stored at -80 ^°^C in a freezer until further use. Franz diffusion cells with a permeation area of 1 cm^2^ were used. The mucosa was removed from the freezer and surgically cut to fit the permeation area of the Franz diffusion cells. Receptor chambers were filled with SNF. The GP-SOL and GP-SLN formulations (each with 50 mg of gabapentin) were then loaded into the donor cell. Studies were carried out at 37 ^°^C with continuous stir at 50 rpm. Samples (1 ml) were collected at specified time points (0, 0.25, 0.5, 0.75, 1, 2, 3, 4, 6, and 8 hr) and an equal volume of fresh SNF was added to maintain sink condition. The permeated amount of gabapentin was determined. Flux (J) was calculated as presented in Equation 3.

$\frac{M}{S}=\mathrm{D.K.Cd}/\mathrm{h.t}$

Equation 3

Where S is the surface area of the membrane (cm^2^) and M represents the amount of gabapentin diffusing through the mucosa (mg). D is the diffusion coefficient of gabapentin (cm^2^/h). K represents the partition coefficient of gabapentin, Cd is the concentration of gabapentin in the donor cell, h is the thickness of the membrane, and t is the time (hour), (n=3, mean±SD).

***Histopathological studies***

To conduct a study on nasal histopathology (37), freshly removed sheep nasal mucosa was obtained from a slaughterhouse. Three nasal mucosa sections of uniform thickness measuring 0.2 mm were selected. The first piece of mucosa was treated with isopropyl alcohol to serve as a positive control, the second piece was treated with phosphate buffer saline (PBS) at pH 6.4 as a negative control, and the third piece was treated with the GP-SLN formulation for one hour. After cleaning with PBS for an hour, the mucosa was immersed in a 10% v/v formalin solution overnight. Each of the three mucosa pieces was placed in a paraffin block before being divided into 5 µm slices using a microtome. To assess any mucosal damage, sections were stained with hematoxylin-eosin and examined under a Zeiss Imager A2 light microscope (Leica, Germany).

***Stability***

After being stored at room temperature (25 ^°^C) for three months, the GP-SLN formulation was analyzed for particle size, polydispersity index, and zeta potential (n=3, mean±SD).

***Statistical analysis***

*The experiments were conducted three times to ensure accuracy (n=3) and the results were used to determine the mean and standard deviation (mean±SD). The GP-SLN formulation was evaluated using the Stat-Ease design of experiment software, and a one-way ANOVA test was applied to confirm the significance with P*<0.05.

## Results

***Box-Behnken design: Preparation and optimization of SLNs***

The Box-Behnken design (BBD) was used to investigate the effects of each factor on the formulations. A three-factor, three-level BBD was performed on various independent factor level combinations, resulting in the 17 formulations shown in Table 2.

Regression from the BBD for each predicted dependent factor is presented in Equations 4-6.

$\mathrm{ps}\left(\mathrm{nm}\right)=214.38+26.85X_{1}-7.44X_{2}-5.39X_{3}-29.2X_{1}^{2}-16.2X_{1}X_{2}-0.65X_{1}X_{3}+14.12X_{2}^{2}-15.83X_{2}X_{3}-5.58X_{3}^{2}$

Equation 4

**PDI**= 0.237+0.014$X_{1}$+0.004$X_{2}$-7.44$X_{2}-5.39X_{3}-29.2X_{1}^{2}-16.2X_{1}X_{2}-0.65X_{1}X_{3}+14.12X_{2}^{2}-15.83X_{2}X_{3}-5.58X_{3}^{2}$

Equation 5

**Zp(mv)**= -237+0.014$X_{1}$-2.26$X_{2}+$0.2$X_{3}$+2.21$X_{1}^{2}$-0.003$X_{1}X_{2}$+3.2$X_{1}X_{3}$+1.26$X_{2}^{2}$-0.45$X_{2}X_{3}$+1.69$X_{3}^{2}$

Equation 65

According to the ANOVA analysis results shown in Table 3, F values greater than 0.05 indicate that the model was significant (38). The Model F values were 4.65, 4.31, and 12.88 for particle size, PDI, and zeta potential respectively. There was only a 2.76% chance for particle size, a 3.36% chance for PDI, and a 0.14% chance for zeta potential that the F value could occur due to noise.

**Figure 1:**
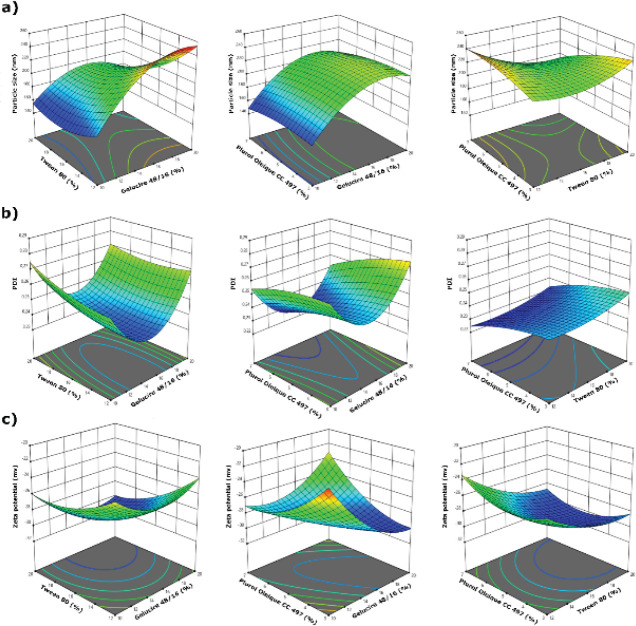
Box-Behnken design (BBD) 3D-graphs showing the effects of independent factor ratios on a) particle size, b) polydispersity index (PDI), and c) zeta potential

The data were imported into the Design Expert software and 3D surface-response graphs were generated, as shown in Figure 1. The impact of independent variables, Gelucire 48/16, Tween 80, and Plurol Oleique on the formulation characteristics, particle size, PDI, and zeta potential were evaluated. Statistical results were consistent with the 3D surface-response graphs.

After conducting the BBD experiments, the desirability point was determined. Based on this, the PDI, zeta potential, and particle size of formulations containing Gelucire 48/16 (15%), Tween 80 (16%), and Plurol Oleique CC 497 (5%) were predicted as 0.237, -29.2 mV, and 214.38 nm, respectively.

*Characterization studies of the GP-SLNs*

Particle size, polydispersity index, zeta potential, and entrapment efficiency

**Figure 2:**
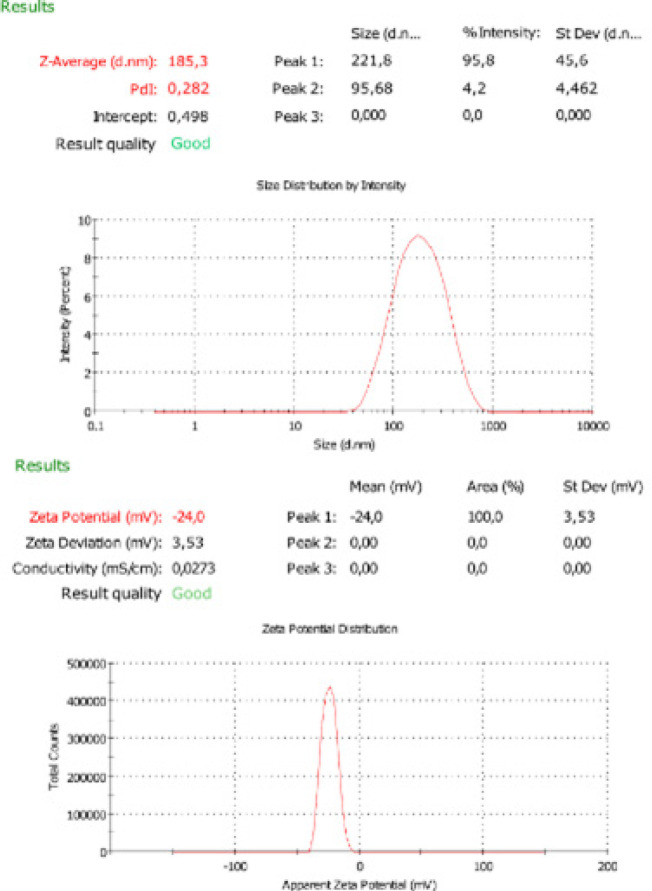
Particle size, polydispersity index (PDI), and zeta potential of the GP-SLN formulation (n=3±SD)

The particle size, PDI, and zeta potential of the optimized GP-SLN formulation were found to be 185.3±45.6 nm, 0.282±0.03, and -24±3.53 mV respectively (Figure 2), (n=3±SD). It was determined that the fabricated GP-SLN formulation was compatible with the desirability point obtained by the BBD. The entrapment efficiency was found to be 82.57±4.02% (n=3±SD). 

*Morphological studies*

**Figure 3:**
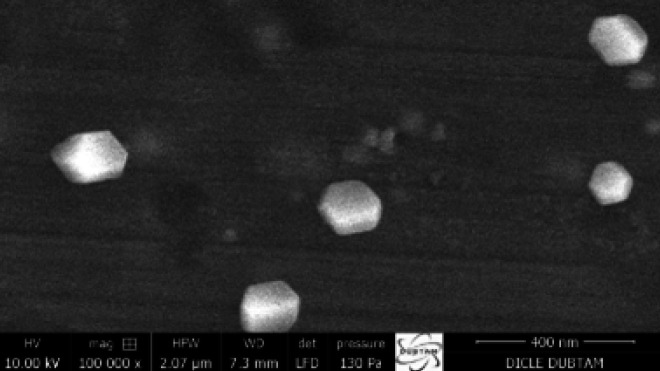
SEM images of the GP-SLN formulation showing cubical shaped nanoparticles

SEM: Scanning electron microscopy; GP-SLN: Gabapentin loaded solid lipid nanoparticle formulation

*The SEM images in* Figure 3 *revealed that the lipid nanoparticles in the GP-SLN formulation had a cubical shape and uniform size distribution. The Zetasizer confirmed the absence of aggregation with a PDI value below 0.3.*

*Solid state characterization studies*

**Figure 4:**
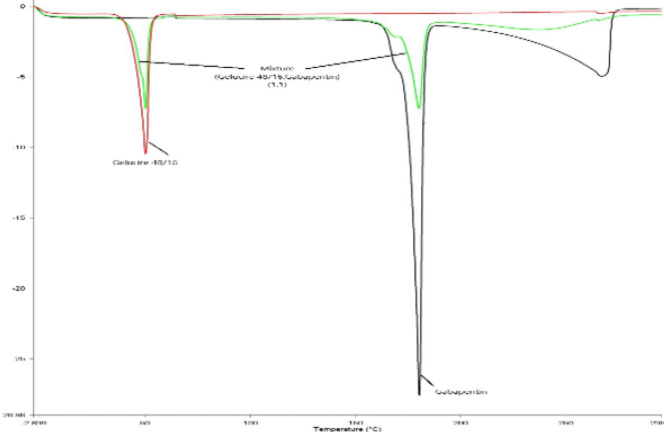
Differential scanning calorimetry (DSC) thermograms of gabapentin, Gelucire 48/16, and physical mixture (1:1)

Figure 4 shows the DSC thermograms of the gabapentin, Gelucire 48/16, and their mixture. The thermogram reveals that gabapentin had an endothermic peak at 166 ^°^C, while Gelucire 48/16 reached an endothermic peak at 50 ^°^C. The individual and mixed peaks suggest that there is no interaction between gabapentin and Gelucire 48/16.

**Figure 5:**
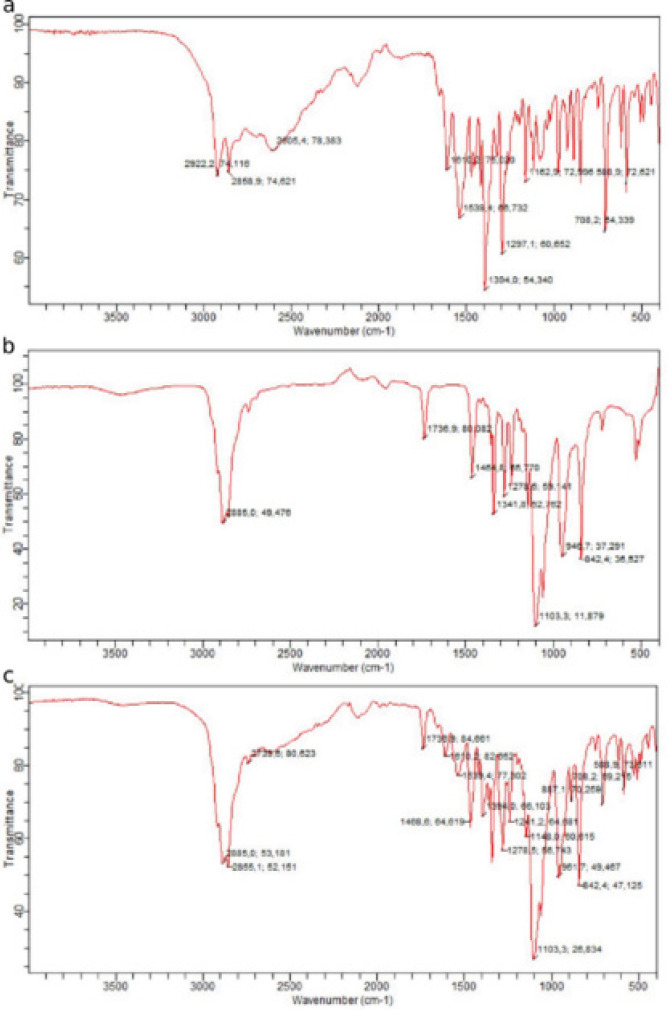
Fourier transform infrared spectroscopy (FTIR) spectra of a) gabapentin, b) Gelucire 48/16, and c) physical mixture

Figure 5 shows the 400 to 4000 cm^-1^ IR range Fourier transform of gabapentin and Gelucire 48/16. Gabapentin’s unique peaks, including the two peaks at 2922 cm^-1^ and 2958 cm^-1^ were stretching vibrations of NH_3_^+^. Peaks at 1539 cm^-1^ were the vibration of NH_3_^+^ deformation and the ionized asymmetric carboxylate group (39). The C=O band at 1810 cm^-1^, the NH_2_ band at 2605 cm^-1^, and the C-N band at 1297 cm^-1^ were revealed (40). Additionally, Gelucire 48/16’s -OH band at 2885 cm^-1^ and the C=O band at 1736 cm^-1^ were also found (41).

After conducting the FTIR experiments on a mixture of gabapentin and Gelucire 48/16, it was found that all major gabapentin-related peaks were present, indicating no interactions between the two compounds.

*In vitro release and release kinetics studies*

**Figure 6:**
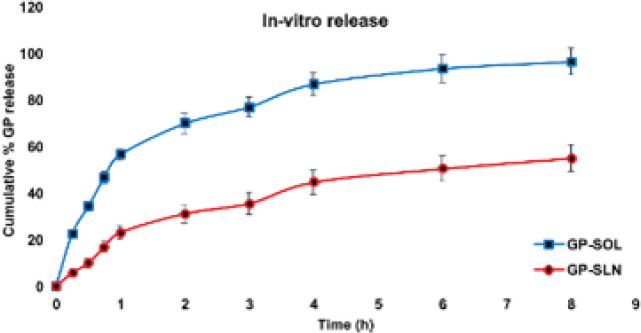
Release profiles of the GP-SOL and GP-SLN formulation (n=3±SD)

GP-SOL: Gabapentin solution; GP-SLN: Gabapentin loaded solid lipid nanoparticle formulation)

**Figure 7:**
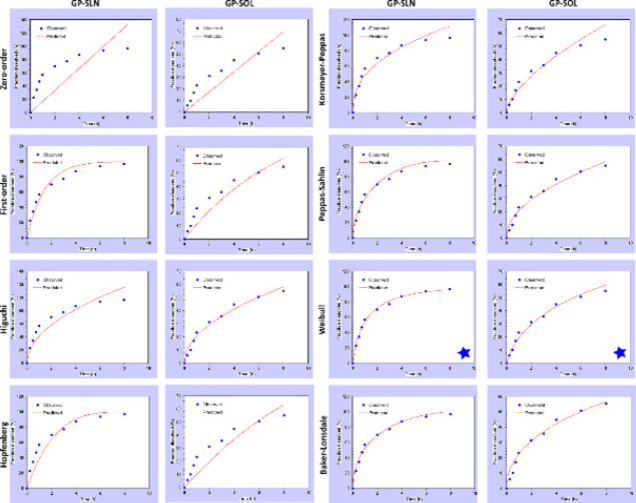
Release kinetics curves obtained with the DDSolver software for the GP-SOL and GP-SLN formulation

GP-SOL: Gabapentin solution; GP-SLN: Gabapentin loaded solid lipid nanoparticle formulation)

In Figure 6, the release of gabapentin from different formulations is presented. The first noticeable effect was a burst release in the GP-SLN formulation, which amounted to 23.164±2.76% after 1 hr. This was followed by a controlled release, with 55.02±5.64% after 8 hr. The release kinetics and models were investigated further, and the results are shown in Figure 7 and Table 4.

Ex vivo permeation studies

**Figure 8:**
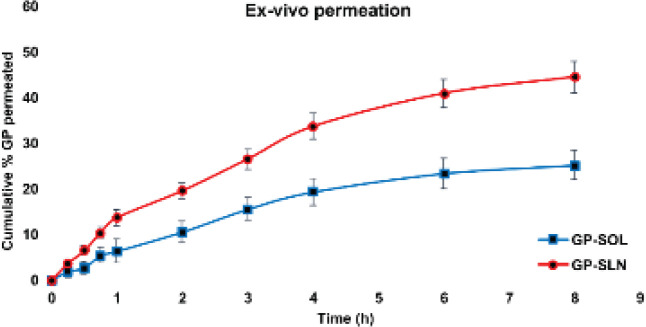
*Ex vivo* permeation of GP from the GP-SOL and GP-SLN formulations (n=3±SD)

GP: Gabapentin; GP-SOL: Gabapentin solution; GP-SLN: Gabapentin loaded solid lipid nanoparticle formulation

The study was conducted on sheep nasal mucosa to investigate the effectiveness of the GP-SLN formulation. The total amount of gabapentin permeated over time was measured and the results showed that the GP-SLN formulation had a higher permeability compared to the GP-SOL. Specifically, 44.45±3.45 mg/cm^2^ of gabapentin was permeated from the GP-SLN formulation, while only 25.178±3.25 mg/cm^2^ was permeated from the GP-SOL. The permeability of the GP-SLN formulation was 1.7 times greater than that of the GP-SOL (Figure 8 and Table 5).

Histopathological studies

**Figure 9:**
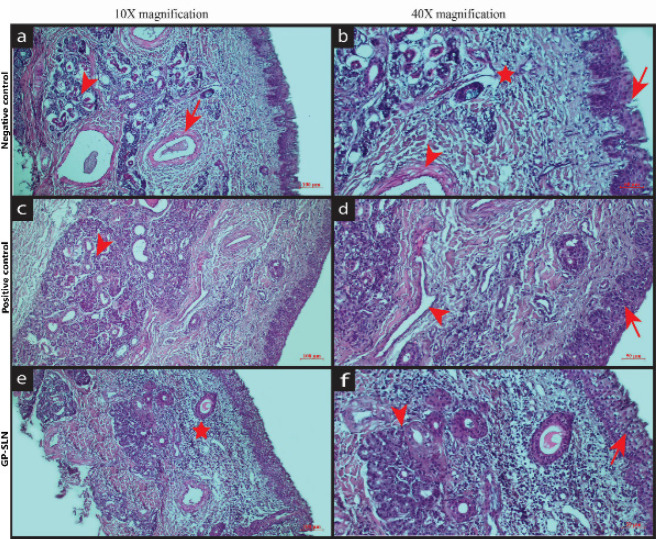
a) Degenerated bowman glands (arrowhead) and internal tissue damage (arrow); b) Degenerated respiratory epithelial cell (arrow), vascular wall (arrowhead), leukocyte infiltration (star); c) Regular glands and ducts (arrowhead); d) Regular lining epithelium of respiratory epithelial cell (arrow), normal vessels (arrowhead); e) Leukocyte infiltration (star); f) Bowman glands (arrowhead), connective tissue and lining epithelium (arrow)

A histopathology study was performed to determine the harmful effects of the excipients utilized in the SLN formulation. Figure 9 shows the histopathological pictures of sheep nasal mucosa. The mucosa treated with isopropyl alcohol (positive control) displayed degenerated bowman glands with a loss of epithelial cells and internal tissue injury (Figures 9a and 9b). When PBS (negative control, Figures 9c and 9d) and the GP-SLN formulation (Figures 9e and 9f) were applied to the nasal mucosa, neither nasociliary damage nor cell necrosis was seen, and the epithelial layer remained intact. The pH value of 6.04±0.45 (n=3±SD) for the GP-SLN formulation was found to be well within the range of human nasal pH (5.5-6.5). These findings suggest that the nasal mucosa was not harmed by the excipients used in the GP-SLN formulation. 

Stability

During the stability study of the GP-SLN formulation, no significant changes in the particle size, PDI, and zeta potential were observed. The formulation retained its physical appearance and the particle size distribution remained narrow (PDI<0.5), indicating that uniform distribution was maintained.

**Table 1:** Dependent and independent variables used in the experimental design to develop solid lipid nanoparticles

Independent factors	Levels		
	Low (-1)	Medium (0)	High (+1)
X_1_ = **Amount of Gelucire 48/16 (%)**	10	15	20
X_2_ = **Amount of Tween 80 (%)**	12	16	20
X_3_ = **Amount of Plurol Oleique CC 497 (%)**	3	5	7
Dependent factors	**Desired**		
Y_1_ = **Particle size (nm)**	Minimum		
Y_2_ = **PDI**	Minimum		
Y_3_ = **Zeta potential (mV)**	Maximum		

**Table 2:** Box-Behnken design: Factors, design, and observed responses to develop solid lipid nanoparticles

Formulations	Independent factors			Dependent factors		
	Gelucire 48/16 (X_1 _%)	Tween 80 (X_2 _%)	Plurol Oleique CC 497 (X_3 _%)	Particle size (nm) (Y_1_)	PDI (Y_2_)	Zeta potential (mV) (Y_3_)	
F1	15	16	5	203.8	0.241	-28.9	
F2	15	20	7	183.7	0.243	-29.6	
F3	15	16	5	242.4	0.235	-28.7	
F4	20	20	5	210.4	0.268	-29.0	
F5	15	20	3	245.6	0.242	-29.4	
F6	15	16	5	210.2	0.238	-29.4	
F7	20	16	3	195.7	0.286	-29.9	
F8	10	16	7	164.8	0.243	-27.1	
F9	20	12	5	256.0	0.268	-24.9	
F10	10	16	3	154.8	0.274	-20.8	
F11	10	12	5	155.8	0.263	-24.0	
F12	15	12	3	230.5	0.232	-23.8	
F13	15	16	5	208.2	0.233	-29.4	
F14	10	20	5	175.0	0.276	-25.0	
F15	20	16	7	203.1	0.245	-23.4	
F16	15	16	5	207.3	0.239	-29.6	
F17	15	12	7	231.9	0.233	-22.2	

**Table 3:** Box-Behnken design: Regression coefficients and *P-values* for dependent factors to develop solid lipid nanoparticles

Coefficients	Responses		
	Particle size (nm)	PDI	Zeta potential (mV)
Standard deviation	17.59	0.011	1.11
Mean	204.66	0.251	-26.77
R-Squared	0.857	0.847	0.943
Model F-value	**4.65**	**4.31**	**12.88**
Model *P*-value	**0.0276**	**0.0336**	**0.0014**

After conducting the BBD experiments, the desirability point was determined. Based on this, the PDI, zeta potential, and particle size of formulations containing Gelucire 48/16 (15%), Tween 80 (16%), and Plurol Oleique CC 497 (5%) were predicted as 0.237, -29.2 mV, and 214.38 nm, respectively

GP-SLN: Gabapentin loaded solid lipid nanoparticle formulation

**Table 4:** Release kinetics modeling and results of the GP-SOL and GP-SLN formulation

Models and equations	Formulation	Evaluation criteria				
		Parameter	r^2^	r^2^ adjusted	MSC	AIC
Zero-order $F=k_{0}*t$	GP-SOL	k_0_ = 16.547	0.186	0,186	-0.445	91.418
	GP-SLNs	k_0 _= 8.663	0,713	0.713	0.796	70.625
First-order $F=100*[1-\exp\left(-k_{0}*t\right)]$	GP-SOL	k_1_ = 0.684	0.961	0.961	2.589	61.078
	GP-SLNs	k_1_ = 0.119	0.862	0.862	1.543	63.336
Higuchi $F=k_{H}*t^{0.5}$	GP-SOL	k_H_ = 40.804	0.891	0.891	1.561	71.362
	GP-SLNs	k_H_ = 20.485	0.977	0.977	3.322	45.370
Korsmeyer-Peppas $F=k_{\mathrm{KP}}*t^{n}$	GP-SOL	k_KP_ = 47.66	0.953	0.948	2.215	64.822
	GP-SLNs	k_KP_ = 17.51	0.938	0.930	2.130	57.287
Peppas-Sahlin $F=k_{1}*t^{m}+k_{2}*t^{2\mathrm{*m}}$	GP-SOL	k_1_ = 60.584	0.986	0.982	3.242	54.553
	GP-SLNs	k_1_ = 19.315	0.974	0.966	2.793	50.66
Weibull $F=100*\{1-\mathrm{Exp}[-({\left(t-\mathrm{Ti}\right)}^{\beta })$**/α]}**	GP-SOL	**β = 0.651**	**0,997**	**0.996**	**4.605**	**40.917**
	GP-SLNs	**β = 0.666**	**0.985**	**0.981**	**3.341**	**45.170**
Hopfenberg $F=100*[1-({1-k_{\mathrm{HB}}\mathrm{*t})}^{n}]$	GP-SOL	k_HB _= 0.101	0.889	0.876	1.351	73.457
	GP-SLNs	k_HB _= 0.035	0.822	0.8	1.075	67.838
Baker-Lonsdale 32*1-1-F1002/3-F100=kBL*t	GP-SOL	k_BL _= 0.061	0.99	0.99	3.941	47.555
	GP-SLNs	k_BL _= 0.009	0.974	0.974	3.182	46.766

GP-SOL: Gabapentin solution; GP-SLN: Gabapentin loaded solid lipid nanoparticle formulation)

**Table 5:** Permeability and flux values of the GP-SOL and GP-SLN formulations (n=3±SD)

	GP-SOL	GP-SLN
Permeability (*P*, cm/h)	0.324 ± 0.035	0.56 ± 0.026
Flux (J, mg/cm^2^/hr)	1.622 ± 0.173	2.801 ± 0.128

GP-SOL: Gabapentin solution; GP-SLN: Gabapentin loaded solid lipid nanoparticle formulation

## Discussion

Recent advancements in nanotechnology have made significant progress in developing sustained-release drugs. The main purpose of the treatment is to target drugs effectively on the tissue, which leads to a decrease in adverse effects and an increase in efficacy. Especially in central nervous system disorders, passing the blood-brain barrier is crucial. As a result, various dosage forms and application techniques are being researched, and nasal delivery of drug molecules is one of the non-invasive techniques under investigation (42, 43). 

Quality issues in pharmaceutical products can often be traced back to the design stage. No matter how much testing is done, a poorly designed product will still be unsafe and/or ineffective. Therefore, it is important to incorporate quality into the product design process itself, rather than relying solely on analysis and testing (44). Traditionally, pharmaceutical products are developed and optimized by analyzing one factor at a time. This involves varying one factor while keeping all others constant. However, this method is time-consuming and does not allow for the evaluation of possible interactions between factors. This can lead to inadequate execution of formulation development and optimization. To overcome these limitations, the design of experiments (DoE) approach can provide better results with a smaller number of experiments. By evaluating multiple factors simultaneously, DoE can help to identify interactions and optimize the formulation development process (45).

In this study, the effects of independent variables on a GP-SLN formulation were analyzed using 3D surface-response graphs, as shown in Figure 1 and Table 3. The particle size of the nanoparticles in the fabricated GP-SLN formulation was in the range of 154.8–256.0 nm. The model was found to be significant, with an f-value of 4.65 and a *P*-value of less than 0.05. According to Equation 4, an increase in the amount of Gelucire 48/16 (X_1_) increased particle size, while an increase in the amount of Tween 80 (X_2_) led to a decrease in particle size as expected. However, excessive use of Tween 80 (X_2_^2^) caused an increase in particle size, which could result in the formation of undesirable structures such as gel or micelle. Similar findings were also observed in different studies (45-49).

PDI was in the range of 0.232 to 0.286. The f-value of the model was 4.31, and the *P*-value was less than 0.05, indicating the significance of the model. The positive sign of X_1_ in Equation 5 suggests that an increase in Gelucire 48/16 leads to an increase in PDI. On the other hand, the negative sign of X_2_X_3_ shows that the amounts of Tween 80 and Plurol Oleique CC 497 lead to a decrease in PDI as expected. In addition, the negative coefficients of X_1_X_2_ and X_1_X_3_ indicate that the use of Gelucire 48/16 with Tween 80 and Plurol Oleique CC 497 leads to a reduction in PDI (45-49). 

The zeta potential was in the range of -20.8 to -29.9 mV. The statistical analysis showed that the model was significant with a high f-value of 12.88 and a *P*-value less than 0.05. Equation 6 revealed that the zeta potential decreased negatively with an increase in the Tween 80 amount. On the other hand, the zeta potential increased due to the positive effect of Plurol Oleique CC 497. The simultaneous use of surfactant and cosurfactants in SLN formulations is crucial as it provides a better steric barrier, which is important for the stability of GP-SLNs (45-49). 

It is essential to ensure high entrapment efficiency of drug delivery systems, which can be challenging when loading a hydrophilic drug into a lipophilic system. However, SLNs have the potential to entrap both hydrophilic and lipophilic drugs (50). This study demonstrated that gabapentin, a hydrophilic drug, was successfully loaded into SLNs at a rate of 82.57±4.02%. 

The therapeutic effects of SLNs depend on the release of drug molecules. However, sometimes the drug molecules stick to the surface of the SLNs and are quickly released into the surrounding medium, which is known as a burst release, which is important for drug delivery systems to maintain a therapeutic concentration for effective treatment (51). In our study, gabapentin was rapidly released in the first hour (23.164%), followed by sustained release (55.02% after 8 hr), which is an example of a burst release (Figure 6). The release of gabapentin from the lipid core took longer than GP-SOL. The structure of the lipid matrix and the concentration of surfactants used can affect the release of gabapentin from SLNs (26). 

Based on the release kinetics parameters displayed in Figure 7, it was concluded that the Weibull model is the most effective for the GP-SLN formulation. This model had the highest values of r^2^, r^2^ adjusted, and MSC, and the lowest AIC values. The “β” parameter in the Weibull model explains the release from the lipid matrix. A “β value” of ≤ 0.75 indicates Fickian diffusion. For the GP-SLN formulation, the β value was found to be 0.666. Mathematical modeling has confirmed that gabapentin released from SLNs is based on Fickian diffusion. Therefore, the GP-SLN formulation can be considered a sustained-release carrier (52). 

According to Table 5, the GP-SLN formulation demonstrated higher flux and permeability compared to the GP-SOL. This suggests that due to their lipophilic structure, the GP-SLN formulation can easily penetrate nasal tissue. Surfactants are also known to improve permeability (26, 42). Gabapentin has a hydrophilic nature and is not able to spread effectively across the nasal membrane. However, when lipophilicity increases, drug delivery systems and drug molecules become more compatible with the nasal membranes. The higher permeability and flux of the GP-SLN formulation can be attributed to its lipophilic nature and nano-sized particles (53). 

## Conclusion

The Quality by Design (QbD) approach is commonly used to fully comprehend the product and manufacturing process. It can also be used to screen and optimize pharmaceutical products and production processes by examining how independent variables affect formulations. By using this, the quality of pharmaceutical products can be improved. In this study, the QbD approach was used as a new concept for fabricating a hydrophilic drug, a gabapentin-loaded SLN formulation. The GP-SLN formulation was created using a modified microemulsion method. The individual effects of CPPs on CQAs were evaluated using the Box-Behnken design to achieve the desired product quality. The optimized GP-SLN formulation was obtained from the overlay plot then it was successfully created with a small particle size (185.3±45.6 nm) and high entrapment efficiency (82.57±4.02%.) The *in vitro* release of the GP-SLN formulation was examined, and it was found that it followed the Weibull kinetic model, with gabapentin release due to Fickian diffusion. It was also shown that SLNs displayed a sustained release. The GP-SLN formulation showed higher drug diffusion (1.7 fold) compared to GP-SOL. The GP-SLN formulation also did not cause any nasociliary damage and/or cell necrosis, indicating that it is safe for nasal administration. From the findings above, it can be concluded that QbD can be successfully applied for the development of an SLN-based colloidal carrier system with fewer trials and higher quality characteristics.

## Authors’ Contributions

MO T and F A designed the experiments; MO T, FA, and MC G performed experiments and collected data; MOT, F A, and MC G discussed the results and strategy; MO T supervised, directed, and managed the study; MO T, F A, and MC G approved the final version to be published.

## Conflicts of Interest

The authors declare no potential conflicts of interest.
